# hMENA splicing program impacts the clinical outcome of early stage lung cancer patients. How and why?

**DOI:** 10.1186/1479-5876-12-S1-P12

**Published:** 2014-05-06

**Authors:** Paolo Visca, Sheila Spada, Francesca Di Modugno, Emilio Bria, Isabella Sperduti, Barbara Antoniani, Gabriele Alessandrini, Belinda Palermo, Vienna Ludovini, Lucio Crinò, Francesco Facciolo, Michele Milella, Marcella Mottolese, Paola Nisticò

**Affiliations:** 1 Regina Elena National Cancer Institute, Rome, Italy; 2 Medical Oncology, Azienda Ospedaliera Universitaria Integrata, University of Verona, Verona, Italy; 3 Medical Oncology, S. Maria della Misericordia Hospital, Perugia, Italy

## Background

In lung cancer, reliable prognostic indicators of the risk of recurrence are still not available. Alternative splicing represents a potential biomarker of diagnosis, prognosis, invasiveness, and response to therapy in different tumors [1], including lung cancer [2].

Human MENA (hMENA) is an actin regulatory protein that modulates cell adhesion and migration [3]. We have isolated three hMENA splice variants, namely hMENA, hMENA^11a^ and hMENAΔv6, impacting differently cell shape and function. hMENA^11a^ expression ensures the integrity of cell-cell adhesion and is associated with an epithelial phenotype, whereas hMENAΔv6 is related to a mesenchymal invasive phenotype. The splicing of hMENA, relevant to epithelial mesenchymal transition, is also regulated by microenvironmental cues [4].

The dynamic reciprocity between tumor and stroma influences the tumor tissue architecture including the T cell localization. This, proposed as a prognostic marker [5], is a prerequisite for antitumor immune surveillance and recently the antibody blockade of immune checkpoints is a new reality in lung cancer treatment [6,7].

## Materials and methods

Pan-hMENA and specific hMENA^11a^ Abs were tested by immunohistochemistry on duplicate TMA from 248 N0 NSCLC, and clinical factors (sex, age, histology, grading, T-size, number of resected nodes, RN) were correlated to 3-yr disease-free (DFS), cancer-specific (CSS), and overall survival (OS) using a Cox model. ROC analysis provided optimal cut-off values and model validation. A logistic equation including regression analysis coefficients was constructed to estimate individual patients’ probability (IPP) of relapse. Internal cross-validation (100 simulations with 80% of the dataset) and external validation was accomplished.

A panel of antibodies recognizing CD3, CD4, CD8, CD20 molecules has been employed for the characterization and localization of lymphocytes, by immunohistochemistry.

## Results

In the series of N0 NSCLC patients (median follow-up: 36 months, range 1-96), Pan-hMENA and hMENA^11a^ were the only biological variables displaying significant correlation with outcome(s), confirmed by the cross-validation (replication rate: 78%, 83%), with a prognostic model accuracy of 61% (standard error 0.04, p=0.0001). The subgroup of patients with High Pan-hMENA/Low hMENA^11a^ relative expression fared significantly better than the other 3 groups (p≤0.002 for all outcomes). On the basis of the combination between this molecular hybrid variable and T-size and RN, a 3-risk class stratification model was generated, discriminating between patients at different risk of relapse, cancer-related death, and death for any cause, with a prognostic accuracy of 61% (standard error 0.03, p=0.01), according to ROC analysis and validated in an independent dataset of 133 patients.

The correlation between hMENA isoforms and the pattern of expression and localization of lymphocytes in the different groups of risk of relapse identified is under evaluation.

## Conclusions

The hMENA splicing program is an early prognostic marker of NSCLC patients and may represent a surrogate marker of a permissive or not tumor microenvironment for lymphocyte recruitment.
